# Use of UV-Polymerizable Methacrylate Adhesive as a Post-Recovery Bandage Replacement for Transphyseal Screw Incisions in Horses: A Pilot Study

**DOI:** 10.3390/ani16193010

**Published:** 2026-09-24

**Authors:** Louise Lafin, Cyril Tricaud, Antoine Lechartier

**Affiliations:** 1 Centre Hospitalier Vétérinaire Equin de Meheudin, 61150 Écouché les Vallées, France; 2 Centre Hospitalier Vétérinaire Equin de Livet, 14140 Saint Michel de Livet, France

**Keywords:** surgical glue, angular limb deformity, surgical site infection

## Abstract

Surgical adhesives may provide an alternative to postoperative bandaging, but their use has not been evaluated in veterinary medicine. This prospective pilot study investigated the tolerance and postoperative adhesion of a UV-polymerizable methacrylate adhesive (UV-PMA) as a post-recovery bandage substitute following transphyseal screw placement in horses. A total of 153 sutured stab incisions from 102 horses were included; 75 received UV-PMA and 78 were protected with bandages. Surgical site infection (SSI) within 30 days and UV-PMA/bandage slippage within 14 days were compared between the treatments for carpal and fetlock incisions using Fisher’s exact tests. For carpal incisions, the SSI rate was significantly different (*p*-value = 0.025), with SSI occurring in 4/31 bandaged incisions (12.9%) and in no UV-PMA incisions. For fetlock incisions, the SSI rates did not differ between groups (3/30 (10%) vs. 6/47 (12.8%); *p*-value = 1.000). The UV-PMA displacement and bandage slippage rates were comparable for carpal incisions (5/45 (11%) vs. 3/31 (10%); *p*-value = 1.000), whereas UV-PMA displacement was significantly more frequent for fetlock incisions (9/30 (30%) vs. 2/47 (4%); *p*-value = 0.003). The UV-PMA shows preliminary potential as a bandage substitute, particularly for carpal incisions, but its higher displacement rate at the fetlock warrants further evaluation in larger randomized studies.

## Simple Summary

Bandages are commonly used to protect surgical wounds in horses, but they require regular replacement and can be difficult to maintain, particularly in young horses. Surgical glue could provide a simpler alternative while allowing wounds to remain visible during healing. This pilot study evaluated a glue that dries under ultraviolet light as a replacement for postoperative bandages in young horses undergoing surgery to correct limb deformities. A total of 102 horses and 153 surgical incisions were included. We compared wound infections and the ability of the glue or bandage to remain in place after surgery. In this study, we did not find evidence that the glue resulted in more wound infections than bandages. Its ability to remain in place depended on the location of the surgical wound. Around the carpus, the glue and bandages had similar rates of displacement, whereas around the fetlock, the glue detached more frequently than the bandages. These preliminary findings suggest that surgical glue may be a potential alternative to bandages for some surgical wounds, particularly around the carpus. Larger studies are needed to confirm its safety and determine where it can be used most effectively.

## 1. Introduction

Surgical glues are bioadhesive materials primarily used in human medicine for organ and skin closure. They have gained significant attention in the medical field as a result of their potential to replace traditional sutures and adhesive wound dressings [1]. Over the past 30 years, their development has focused on requirements that include easy application within a short time, the ability to polymerize in a moist environment, and adequate tensile strength. Acrylate-based monomers exhibit high tensile strength, and resistance to both deformation and humidity [2]. Therefore, they offer superior adhesive properties compared to traditional rubber-based adhesives commonly used in the treatment of human wounds [1,3]. Methacrylate forms a transparent, waterproof film on the skin surface, protecting wounds from external contamination and enabling continuous inspection of the healing process [2].

In human medicine, several studies have evaluated acrylate-based monomers as surgical adhesives, either as alternatives or adjuncts to sutures and dressings, demonstrating benefits such as reduced bandage changes and earlier rehabilitation [2,4,5,6]. In contrast, the evidence remains limited in veterinary medicine. Only a few studies have investigated surgical adhesives for incisional wound closure, including one in dogs [7] and one in horses in which the surgical incisions were still covered with bandages [8]. Three reports have evaluated the use of UV-polymerizable methacrylate adhesive (UV-PMA) in horses as the second layer of ex vivo jejunal anastomosis [4], for second-intention healing of naturally occurring wounds [5] and for closing exploratory laparotomy surgical wounds [6]. Therefore, to our knowledge, no veterinary studies have evaluated the use of UV-PMA or any surgical adhesive as a substitute for bandaging after surgical wound closure.

Transphyseal screws are commonly placed in juvenile horses to correct angular limb deformities, typically between 1 and 3 months of age for the fetlock and up to 18 months for the carpus, although the exact physeal closure varies among individuals [7]. This procedure may be performed at several anatomical sites in the same individual, including the distal aspect of the third metacarpus or the distal radius. Reported surgical site infection rates after transphyseal screw placement range from 3% to 8% [8,9,10]. Transphyseal screw insertion requires only a single stab incision closed with one interrupted suture [11]. It therefore provides a practical and standardized model for assessing surgical glue as a potential alternative to postoperative bandaging. This procedure offers good repeatability of wound closure and easy monitoring of wound healing. Sterile gauze and elastic adhesive tape are commonly used to protect the surgical site for 10 to 15 days until suture removal. However, maintaining bandage integrity and position can be challenging, particularly in young horses, and complications such as pressure sores may occur [12]. Therefore, an alternative to traditional bandaging may be beneficial.

The objective of this pilot study was to investigate the skin tolerance of a UV-polymerizable methacrylate adhesive (UV-PMA) as a bandage substitute for transphyseal screw wounds after surgical closure. We hypothesized that (1) UV-PMA would not lead to more surgical site infections (SSIs) and that (2) UV-PMA would provide sufficient postoperative adhesion, with a displacement rate comparable to the bandage slippage rate.

## 2. Materials and Methods

### 2.1. Study Design

This study was conducted as a prospective exploratory study, and included all available individuals who met the inclusion criteria. All the horses included in the study were presented for transphyseal screw placement to correct angular limb deformity at two equine referral centers between April 2024 and April 2025. All the horses were client-owned, and informed consent was obtained prior to surgery. Horses with multiple angular limb deformities were included. All the animals underwent a routine preoperative examination, which included a clinical examination, hematology, and biochemistry. A careful skin inspection at the proposed surgical site was performed. Animals with pre-existing skin lesions at the proposed surgical site, such as pressure sores from previous bandaging or superficial wounds sustained during transport or in a paddock, were excluded. Animals whose bandage or glue application was not performed according to the established protocol and those with incomplete follow-up were excluded. Based on the owners’ consent, obtained prior to surgery, the horses were either randomly assigned to a group using a coin toss or directly assigned to either the bandage group or the glue group. The owners of 56 horses consented to random treatment allocation. Among those, 21 horses with carpal incisions were allocated to the UV-PMA group and 12 to the bandage group, while 16 horses with fetlock incisions were allocated to the UV-PMA group and 7 to the bandage group. Therefore, only partial randomization was achieved. Approval from the ethics committee was obtained on 28 March 2024 (Comité d’Ethique de VetAgro Sup n°18, Lyon, France; approval number 2414).

### 2.2. Procedures

The standard clinical protocols for both practices were used for this procedure, including antibiotic prophylaxis, anesthesia, and all perioperative management, and no aspects were modified specifically for the purpose of this study. All surgical procedures were performed by three surgeons following the technique described by Kay and Hunt (2009) [13]. Premedication consisted of butorphanol (Butador, Boehringer, Ingelheim am Rhein, Germany; 0.05 mg/kg, i.v.) and romifidine (Rominervin, Dechra, Montigny le Bretonneux, France; 0.05 mg/kg, i.v.). Anesthesia was induced with ketamine (Ketamidor, Axience, Pantin, France; 2.2 mg/kg, i.v.) and diazepam (Diazedor, Axience, Pantin, France; 0.08 mg/kg, i.v.) and maintained with isoflurane (Isorane, Axience, Pantin, France) in oxygen. Continuous rate infusions of dobutamine (Dobutamine, Mylan, Lyon, France), 0.5–2 µg/kg/min IV, were administered throughout surgery. Perioperative antibiotic prophylaxis was provided with a single dose of trimethoprim–sulfadiazine (Borgal, Virbac, Carros, Biot, France) of 15 mg/kg IM, while flunixin meglumine (Genixine, Ceva, Nice, France) at 1.1 mg/kg IV was administered as an anti-inflammatory. Trimethoprim sulfate was administered intramuscularly, as this route is licensed for use in horses and avoids the intravenous administration of potentiated sulfonamides, which is contraindicated in horses sedated with α2-adrenergic agonists because of the risk of potentially fatal cardiac dysrhythmias. The skin was clipped with a 0.25 mm clipper blade and aseptically prepared with three consecutive rounds of povidone iodine soap and isopropyl alcohol, ending with a povidone iodine spray. Adhesive draping was used to delineate the surgical site. At the end of surgery, each stab incision was closed with a single horizontal mattress suture using 3-0 polyglactin 910 (Vicryl Rapid, Ethicon, Issy les Moulineaux, France). The wound was dried with sterile gauze.

For the horses in the UV-PMA group, a small drop of approximately 1 mL of UV-PMA glue (Cohesives Laboratories, Dijon, France) was placed on a sterile plastic spatula and applied in a thin layer over the wound in all four directions according to the manufacturer’s instructions, to obtain a uniform layer without streaks. The glue was polymerized with an LED UV lamp (Cohesives Laboratories, Dijon, France) for 60 s, at a maximum distance of 10 cm, and the process was repeated for a second layer (Figure 1). Once fully dry, a single-layer bandage consisting of sterile surgical cotton (Coton gaze HM, Horse Master, Paris, France), Velpeau crepe band (Lohmann & Rauscher, Remiremont, Germany), and self-adherent cohesive wrap tape (Flex-on, Horse Master, Paris, France) was applied to the limb for recovery and removed once the horse was standing.

In the control group, a single-layer bandage consisting of non-adherent cotton–polyester dressing (Melolin, Smith & Nephew, Neuilly sur Seine, France), sterile viscose cast padding (BSN Medical, Vibraye, France), sterile surgical cotton gauze (Coton gaze HM, Horse Master, Paris, France), Velpeau crepe band (Lohmann & Rauscher, Rengsdorf, Germany), self-adherent cohesive wrap tape (Flex-on, Horse Master, Paris, France) and elastic adhesive bandage (Tensoplast Vet, BSN Medical, Vibraye, France) was applied. The carpal bandages extended from the mid-radius to the mid-third metacarpus, whereas the fetlock bandages were applied as half-limb bandages. Bandages were replaced 24 h after surgery prior to discharge.

### 2.3. Postoperative Management

Foals were discharged the day after surgery. At discharge, the horses in the UV-PMA group had surgical glue on their stab incisions without bandages, whereas the horses in the bandage group had their stab incisions covered only with a bandage. All horses were prescribed a four-day course of oral meloxicam (Rheumoxydil, Audevard, Clichy, France; 0.6 mg/kg, SID), and no postoperative antibiotic treatment was administered. Until the sutures were removed, the horses were monitored once daily by farm managers. Monitoring included daily bandage/glue integrity, temperature and lameness checks. In the event of any lack of bandage/glue, fever (rectal temperature > 38.2 °C) or lameness in operated limbs, the farm managers were asked to notify the hospital and to request a visit on the same day. Horses were kept stalled until the sutures were removed. These recommendations were clearly stated on the discharge note. In the UV-PMA group, the surgical glue and wound healing were monitored at the stud by a veterinarian on day 7 and day 14 after surgery until the sutures were removed. All postoperative procedures, including bandage changes, UV-PMA renewal, wound inspections, suture removal, and follow-up evaluations, were performed either by the operating surgeon or by another veterinarian from the hospital team. Sutures were removed under aseptic conditions 14 days after surgery. Following suture removal, the same recommendations for daily lameness and temperature monitoring as those provided at discharge were reiterated and documented on the prescription note to continue until day 30. The horses were then re-evaluated weekly at the stud by a veterinarian to assess the limb angle and determine the appropriate time for screw removal.

For the bandage group, the dressing was changed 7 days postoperatively and maintained until suture removal on day 14. At each bandage change, the surgical wounds were thoroughly inspected. In the absence of discharge, a sterile bandage was reapplied using the same standardized protocol as described intraoperatively. In both the UV-PMA and bandage groups, any premature displacement of the bandage or adhesive prior to suture removal prompted wound inspection, disinfection, and replacement of the bandage or UV-PMA on the farm by a veterinarian using the same protocols as previously described. UV-PMA was reapplied after displacement only when no wound discharge was present.

### 2.4. SSI Management

In both groups, when swelling and/or discharge was present, the wound was carefully inspected by a veterinarian from the corresponding surgical team. The extent of the infection was assessed by ultrasonographic and radiographic examination of joint and associated soft tissues, confirming its localization to the subcutaneous tissues and excluding deeper involvement. Surgical site infections were therefore considered superficial according to the criteria described recently in [14]. The wound was cleaned three times with povidone–iodine soap and isopropyl alcohol, and the bandage was then replaced in the same manner as previously described. In the event of an SSI from a stab incision in the UV-PMA group, the glue was gently removed with isopropyl alcohol and soap. The incisional wound was cleaned three times following the protocol described above and then covered by a bandage. As a first-line treatment for SSI, horses received a 7-day course of oral trimethoprim–sulfadiazine (Equibactin, Dechra, Montigny le Bretonneux, France) at a dose of 30 mg/kg. Horses presenting with an SSI had their bandages replaced by a veterinarian from the hospital every 48 h until the infection was resolved. Bacteriological sampling was performed only in cases where infection persisted after two bandage replacements despite systemic antimicrobial therapy.

### 2.5. Outcomes Assessment

The primary outcome was defined as the presence or absence of skin infection within 30 days following surgery. A skin infection was defined as any subcutaneous or cutaneous swelling associated with purulent exudate draining from the surgical wound [14]. The secondary outcome recorded was the presence of bandage slippage or glue displacement during the first 14 days post-surgery. Bandage slippage was defined as slippage of the bandage resulting in exposure of the surgical incision, while UV-PMA displacement was defined as partial or complete detachment of the adhesive resulting in exposure of the surgical incision. Wound monitoring was conducted by seven veterinarians, including one of the surgeons. Neither the observers nor surgeons were blinded to the treatment allocation. In the bandage group, the bandage integrity and wound healing were monitored at the stud by a veterinarian on day 7 and day 14 after surgery until the sutures were removed. In the UV-PMA group, the surgical glue and wound healing were monitored at the stud by a veterinarian at the same intervals. During each evaluation, the wounds were carefully inspected by the veterinarian, and any pressure sores or alopecic areas or skin reactions were noted. Upon suture removal, wound evaluation was performed weekly at the stud, concurrently with angular limb deformity examination.

### 2.6. Data Analysis

A sample size calculation was performed to determine the number of surgical wounds needed for testing the non-inferiority of UV-PMA compared to bandages. The infection prevalence in the control group following a transphyseal screw procedure was estimated at 5% (p_1_ = 0.05), based on infection rates reported in the literature [13]. To detect an increase in the infection rate in the UV-PMA group of 5 percentage points (p_2_ = 0.10), with a power of 80% and statistical significance set at α = 0.05, a sample size of 434 wounds per group was required. The variables (horse, age, breed, sex, date of screw implantation/removal, group assignment, bandage/glue displacement and skin infections) were recorded throughout the study period and compiled in an Excel spreadsheet (Microsoft Excel, version 16.102) for each surgical wound. No missing data were encountered. Descriptive statistics were compiled for age, sex, breed, angular limb deformity (ALD) type and joint distribution in both groups. Breakdown comparisons of sex, breed and ALD type between the two groups were evaluated using Pearson’s Chi-squared test or Fisher’s exact test as appropriate. The age distribution in each group was tested for normality using a Shapiro–Wilk test and compared between the two groups using a Wilcoxon rank-sum test. Age was compared between the two anatomical sites using a Wilcoxon rank-sum test. Comparisons of infection rate and glue/bandage slippage between the groups for each joint were performed with Fisher’s exact test. Associations between the explanatory variables and the outcomes were expressed as odds ratios (ORs) with corresponding 95% confidence intervals (CIs). Statistical significance was assessed using two-sided tests, with a *p*-value < 0.05 considered statistically significant. Statistical significance was set at *p* < 0.05. All analyses were performed in Rstudio (R version 4.5.0 (2025-04-11)) [15]. Packages including ggplot2 [16] for figure generation were also used.

## 3. Results

### 3.1. Descriptive Analysis and Data Repartition

A total of 153 stab incisions from 102 horses were included. Seventy-five sutured stab incisions (48 horses on 45 carpi and 30 fetlocks) received UV-PMA glue, while 78 sutured stab incisions (59 horses on 31 carpi, 47 fetlocks) were protected with bandages and served as the controls. The planned sample size determined by the power analysis was not reached. Based on the observed SSI rates of 4.0% (3/75 incisions) in the UV-PMA group and 12.8% (10/78 incisions) in the bandage group, a post hoc power analysis using the actual sample sizes yielded an estimated statistical power of approximately 53% at a two-sided α level of 0.05. Patient enrolment is summarized in Figure 2. The results are presented as the mean ± standard deviation (SD) for the normally distributed data or as the median (first quartile [Q1], third quartile [Q3]) for the non-normally distributed data, as appropriate.

Overall, the study included 102 horses with a median age of 2 months (Q1, Q3: 1, 10 months). Forty-five horses (44%) were male and 57 (56%) were female. The breeds included 65 Thoroughbreds (64%), 29 Standardbreds (28%), four Arabians (4%), and four Warmbloods (4%). Among the surgical wounds, 68 varus deformities (44%) and 85 valgus deformities (56%) were recorded. In the bandage group, 32 wounds were associated with varus and 49 with valgus deformities; in the UV-PMA group, 36 were associated with varus and 39 with valgus deformities. The median age was 56 days (Q1, Q3: 42, 67) for fetlock deformities and 326 days (Q1, Q3: 127, 383) for carpal deformities. In total, the affected joints included 76 carpi and 77 fetlocks. There were no differences between groups in terms of sex, breed, or deformity distribution. The horses in the bandage group were younger compared to those in the surgical glue group (Estimate = −1; 95% CI: −2 to <0.001; *p*-value = 0.007), and the joints were not evenly distributed between the groups, with a higher proportion of carpi in the glue group (Estimate = 8; 95% CI: 7.9 to 9; *p*-value = 0.019). Distribution of variables between the bandage and glue groups is summarized in Table 1. The joint involved was associated with the age of the horse (*p*-value < 0.001), with fetlock joints operated on much earlier (56 days (Q1, Q3: 42, 67)) than the carpi (326 days (Q1, Q3: 127, 383)).

### 3.2. Surgical Site Infection Rate

Postoperative skin infections occurred in 13/153 stab incisions (8.5%) in 10/102 (10%) horses. Those SSIs included three fetlocks (in 2/45 horses (4%)) in the UV-PMA group and six fetlocks (in 5/57 horses (9%)) and four carpi (in 3/57 horses (5%)) in the bandage group. For the carpal incisions, no SSI was recorded in the UV-PMA group. The number of SSIs reported in each group is represented in Table 2.

For the carpal incisions, the difference in the SSI rate was significant (OR = 0; 95% CI: 0–0.99; *p* = 0.025) between the UV-PMA and bandage groups, with the bandage group showing a higher SSI rate than the carpus. For the fetlock incisions, the SSI rates did not differ significantly between groups (95% CI: 0.11–3.94; *p*-value = 1.000). The number of infections for each joint between groups is represented in Figure 3.

All infections were observed between day 5 and day 15 after surgery, with a majority (8/13 wounds, in six horses) occurring after 10 days. All postoperative infections resolved within 7 days following repeated wound cleansing every 48 h using povidone–iodine soap and isopropyl alcohol, combined with bandage replacement and oral administration of trimethoprim–sulfadiazine (Equibactin, Dechra, Montigny le Bretonneux, France) at 30 mg/kg. All infections were subcutaneous and did not lead to implant infection. No skin reactions secondary to UV-PMA application were noted.

### 3.3. UV-PMA Adhesion/Bandage Slippage

UV-PMA displacement or bandage slippage occurred in 19/153 (12%) incisions in 12/102 (12%) horses during the first 14 days postoperative. For carpal incisions, UV-PMA displacement occurred in 5/45 (11%) incisions, and bandage displacement occurred in 3/31 (10%). This difference was not statistically significant (OR = 1.16; 95% CI: 0.21–8.11; *p*-value = 1.000). For fetlock incisions, UV-PMA displacement occurred in 9/30 (30%) incisions, compared with bandage displacement in 2/47 (4%). This difference was statistically significant (OR = 9.35; 95% CI: 1.72–96.37; *p*-value = 0.003). Overall, the UV-PMA remained in place without requiring renewal in 61/75 (81%) incisions. No SSI developed following UV-PMA renewal after displacement, whereas three of the five incisions with bandage slippage developed a subcutaneous infection.

## 4. Discussion

To the best of our knowledge, this pilot study is the first study to investigate the suitability of UV-PMA as a substitute for post-recovery bandaging following transphyseal screw placement in horses. Few studies in veterinary medicine have investigated surgical adhesives for incisional wound closure or second-intention wound healing [7,8,10], and none have explored their use as a potential substitute for post-recovery bandaging after wound suturing, as the animals in these studies still received a postoperative bandage. Direct comparison of our findings with those of previous veterinary studies is therefore limited.

The first hypothesis was partly supported, as the UV-PMA was not associated with a higher SSI rate than bandaging at either anatomical site. However, the small sample size compared to the a priori calculations makes this study greatly underpowered, which is reflected by the large confidence intervals and does not allow for a definitive conclusion about this hypothesis. The evidence from human surgeries regarding the effect of cyanoacrylate tissue adhesives on SSIs remains inconsistent, with no clear evidence that their additional use reduces SSI rates [2]. In a randomized controlled trial of 105 patients undergoing acetabular fracture repair with staple skin closure, the addition of cyanoacrylate adhesive did not significantly reduce SSIs compared with conventional wound dressings [17]. Prospective non-randomized studies in soft-tissue surgery have yielded conflicting results. In a cohort of 450 patients undergoing abdominoplasty, the use of cyanoacrylate adhesive was associated with an absolute reduction in SSI risk of 2.7% [18], whereas no SSI occurred in either the cyanoacrylate or control group in a cohort of 190 pediatric patients [19]. Finally, one retrospective study showed that SSI rates differed significantly in a cohort of 2100 patients, with a higher infection rate in patients receiving cyanoacrylate adhesives than in those managed with conventional wound dressings [20]. Overall, the current evidence from human surgeries does not demonstrate a consistent effect of cyanoacrylate adhesives on SSIs. Although these findings cannot be directly extrapolated to horses or to UV-PMA adhesives, the inconsistent evidence regarding SSI prevention, together with the practical advantages associated with tissue adhesives, warrants further investigation of their use as alternatives to conventional postoperative dressings. In horses undergoing ventral midline celiotomy, similar surgical site infection rates were reported following skin closure with n-butyl cyanoacrylate and stainless steel staples (11.0% and 12.4%, respectively), with no significant difference between closure methods [7]. Similarly, another study found no significant difference in surgical site infection rates following closure with 2-octyl cyanoacrylate mesh, metallic staples, or sutures [19]. Therefore, although cyanoacrylate-based adhesives have been proposed to provide a barrier against bacterial contamination, the current evidence in horses does not demonstrate a significant reduction in surgical site infections associated with their use. This may be particularly relevant following transphyseal screw placement in foals, for which maintaining a clean and dry surgical site has been considered important for reducing the risk of SSIs [21,22]. In addition, using surgical glue eliminates the need for a postoperative bandage and could avoid problems associated with bandage slippage, such as pressure sores, which are a main concern in juvenile horses [12]. Furthermore, an earlier return to controlled exercise rather than prolonged stall confinement has been advocated following transphyseal screw placement to limit excessive limb laxity [23]. Protecting the surgical wound without maintaining a limb bandage could therefore be advantageous during this early rehabilitation period. Similar practical benefits have been reported in human medicine, where tissue adhesives have been associated with fewer dressing changes and earlier rehabilitation [2].

The infection rate in our study was 8.5% (13/153 incisions in 10/102 horses), which is higher than the rates previously reported following transphyseal screw placement in horses [8,9,10]. Several factors may have contributed to this finding. The perioperative antimicrobial protocol consisted of a single dose of trimethoprim–sulfamethoxazole (30 mg/kg) before induction, with no routine postoperative antimicrobial treatment, and the potential influence of this protocol on SSI occurrence cannot be excluded. Antimicrobial prophylaxis and postoperative antimicrobial use were not reported in previous studies describing transphyseal screw placement [8,9,10,13], precluding direct comparison of antimicrobial protocols and their potential influence on SSI rates. Nevertheless, none of the SSIs involved the implants, and all resolved following local wound management and antimicrobial treatment in accordance with the information reported in the literature [21]. The prospective design and regular postoperative monitoring may also partly explain the relatively high SSI prevalence. The suture material and closure pattern should also be considered when interpreting the SSI rate observed in the present study. Polyglactin 910, a braided multifilament absorbable suture material, was used for skin closure in accordance with our hospital’s routine clinical protocol for transphyseal screw placement and arthroscopy in young foals and yearlings. This approach is routinely used in this population because it facilitates suture removal with minimal manipulation and restraint, thereby simplifying postoperative management and reducing handling-related risks for both the animal and veterinary personnel. Nevertheless, the suture material and pattern have been identified as factors potentially influencing the development of SSIs in horses, and the use of polyglactin 910 has previously been associated with an increased risk of SSI in equine surgery [24]. Furthermore, the horizontal mattress pattern used for skin closure may result in local tissue compression when excessive tension is applied, potentially compromising tissue perfusion and wound healing [25]. Farm managers were instructed to conduct daily lameness and temperature monitoring on the discharge note. Increased rectal temperature (>38.2 °C) was the most frequently reported clinical sign prompting veterinary evaluation. In addition, the horses underwent regular veterinary follow-up during the first month postoperative. This regular surveillance may have facilitated the detection of minor superficial or subcutaneous infections that may have been underreported in previous retrospective studies, in which only the cases with the most severe clinical signs of infection may have been detected and recorded. The transparency of UV-PMA also allowed for direct inspection of the surgical incision without dressing removal, potentially further increasing the detection of minor wound complications in this group. No adverse skin reactions associated with the UV-PMA application were observed in the present study. Similarly, no specific adverse skin reactions attributable to cyanoacrylate adhesives were reported in previous studies evaluating n-butyl cyanoacrylate or 2-octyl cyanoacrylate mesh for skin closure following ventral midline celiotomy in horses [7,19]. Although these products differ from the UV-PMA used in the present study, these findings suggest good local tolerance of these adhesive materials in horses.

Fetlock incisions appeared to be associated with a higher number of SSIs than carpal incisions. The proximity of the fetlock to the ground and its greater exposure to environmental contamination could have potentially contributed to this finding. However, to our knowledge, site-specific SSI rates following transphyseal screw placement at the carpus and fetlock have not been reported. Nevertheless, anatomical location may influence infection risk. Previous studies have demonstrated site-related differences in equine skin bacterial flora and bacterial reduction following aseptic preparation [26], and the joint treated has been identified as a risk factor for postoperative septic arthritis following elective arthroscopy [27]. These findings suggest that differences in the local bacterial burden and other site-specific factors could potentially contribute to differences in SSI risk between anatomical locations. However, whether such differences exist specifically between carpal and fetlock transphyseal screw incisions remains unknown. A significant favorable difference in the SSI rate was observed for carpal incisions in the UV-PMA group compared with the bandage group; however, this finding should be interpreted with caution given the wide confidence interval, limited statistical power, and potential influence of unaccounted confounding factors.

Regarding the second hypothesis, the ability of UV-PMA to maintain coverage of the surgical incision appeared to depend on the anatomical location. Among the carpal incisions, UV-PMA displacement occurred in 5/45 (11%) incisions, compared with bandage slippage in 3/31 (10%), with no significant difference between treatments. Conversely, at the fetlock level, UV-PMA displacement occurred in 9/30 (30%) incisions compared with bandage slippage in 2/47 (4%), representing a significant difference between treatments. Thus, our second hypothesis was not supported, particularly for fetlock incisions. This finding contrasts with the reportedly high adhesion of methacrylate-based surgical glue [1]. However, adhesive strength alone may not determine its ability to remain in place. Once polymerized, UV-PMA forms a relatively stiff layer with limited ability to accommodate substantial tissue deformation, as previously observed experimentally [4]. On a distal limb, repeated joint motion and the associated deformation of the skin may therefore generate mechanical stresses at the adhesive–skin interface, potentially contributing to progressive lifting or detachment of the glue despite its high initial adhesion. The poorer performance at the fetlock may be related to its anatomical location and greater exposure to movement, friction, and environmental conditions. Most UV-PMA displacement events (12/14) occurred during the first six months of the study. As the UV-PMA was a novel technique for the participating surgeons, an initial learning curve and suboptimal application technique may have contributed to these early failures. In particular, adequate drying of the surgical site may be critical for optimal adhesion, as acrylate bonding relies partly on the penetration into surface irregularities and subsequent interlocking with the tissue, and therefore requires application onto a strictly dry surgical site [1]. Therefore, insufficient drying of the surgical site, particularly during the initial learning period, may have contributed to some of the observed UV-PMA displacement events. Bandage displacement was more frequent for the carpus than fetlock (10% vs. 4%). This difference may be partly related to the distinct biomechanics of these joints. Range of motion was reported at approximately 68.7 ± 5.7° for the carpus and 60.2 ± 1.4° for the fetlock, corresponding to a difference of approximately 8° [28]. This suggests that factors other than joint mobility alone, such as regional anatomy, bandage conformity, and sub-bandage pressure, may play an important role in bandage stability [29]. In this regard, substantially higher sub-bandage pressures for fetlock bandage configurations than for carpal elastic bandages have been reported [26], which may contribute to improved bandage stability at the fetlock. Importantly, no SSI developed following UV-PMA renewal after displacement. Reapplication of the adhesive may have restored a physical barrier against environmental contamination of the surgical incision. Antimicrobial activity has also been described for some tissue adhesives, particularly cyanoacrylate-based products [2], and could theoretically contribute to this effect. However, such properties have not been specifically demonstrated for the UV-PMA adhesive evaluated in the present study, and this hypothesis therefore requires further investigation. Nevertheless, the relatively high displacement rate at the fetlock represents an important practical limitation, as the need for reapplication reduces the potential advantages of UV-PMA as an alternative to conventional bandaging.

Several limitations should be considered when interpreting the results of this study. First, this was a pilot study, and the final sample size was smaller than that initially planned, despite predictions based on the annual caseloads of both participating centers. Consequently, the study was underpowered to detect the initially hypothesized effect. Although a significant difference in the SSI rate was identified for carpal incisions, the wide confidence interval indicates considerable uncertainty regarding the magnitude of this association. Similarly, the absence of a significant difference at the fetlock should not be interpreted as evidence of equivalence between treatments. Both results require confirmation in a larger study with sufficient statistical power to provide more precise estimates of treatment effects. A major limitation was the incomplete randomization of treatment allocation. Owners who consented to random treatment allocation had their horses randomly assigned, whereas some studs were reluctant to use UV-PMA and others specifically requested it, resulting in direct treatment assignment for part of the study population. This may have introduced allocation bias and contributed to imbalances in the anatomical site and other potential confounding factors between treatment groups. The uneven distribution of anatomical sites between treatment groups represents another important limitation of our study. Due to the lack of randomization, the two groups did not have an even distribution of cases regarding the joint involved. Stratification of the analyses by anatomical site was therefore used to reduce the influence of the marked imbalance between carpal and fetlock incisions. However, this approach further reduced the sample size available for each comparison and could not account for other potential confounding factors. In particular, it did not account for clustering of multiple incisions within individual horses or the potentially uneven distribution of stud farms or surgeons between groups. Therefore, our results should be interpreted cautiously and require confirmation. Finally, the postoperative environmental management was not standardized among stud farms, including housing conditions, stable size, bedding material, and cleaning practices. Variability in these factors may have influenced surgical site contamination and wound healing, and therefore represents a potential source of unmeasured confounding.

## 5. Conclusions

In conclusion, this first pilot study provides preliminary evidence supporting the feasibility of using UV-PMA as an alternative to postoperative bandaging following transphyseal screw surgery, particularly for carpal incisions. The UV-PMA was not associated with a higher SSI rate than bandaging at either anatomical site; however, its adhesive performance was site-dependent, with a significantly higher displacement rate than bandage slippage at the fetlock level. These findings should be interpreted with caution, as the study was markedly underpowered, not randomized, and therefore may have lacked sufficient statistical power to detect clinically relevant differences between treatment groups. Larger, fully randomized studies with balanced treatment allocation and statistical methods accounting for repeated observations within horses are warranted to confirm these findings and further evaluate the use of UV-PMA as a postoperative bandage substitute in equine surgery.

## Figures and Tables

**Figure 1:**
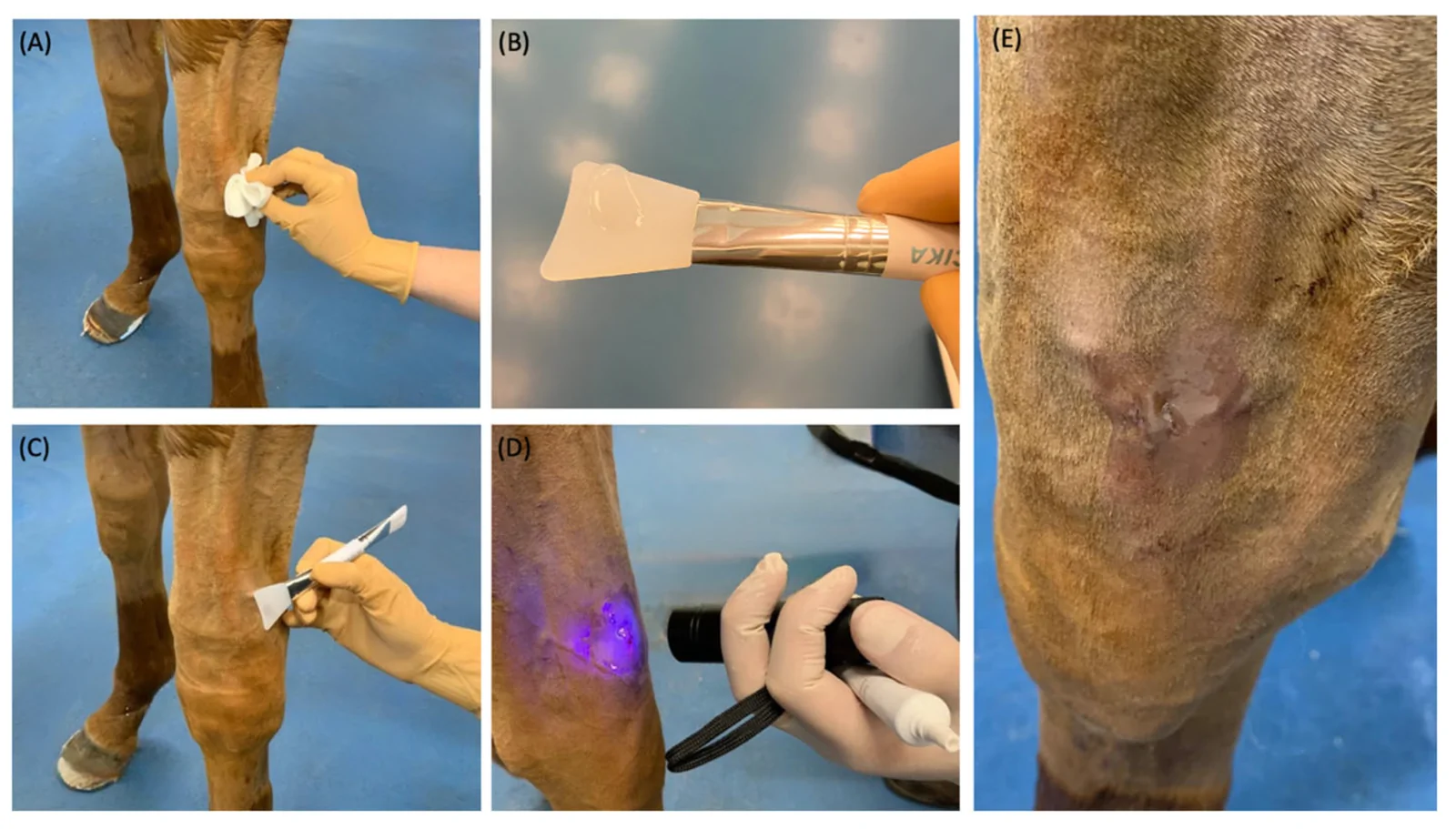
Representative images demonstrating the UV-PMA glue application protocol in a standing horse following transphyseal screw implantation at the lateral aspect of the left distal radial physis. The application steps shown are identical to those performed under general anesthesia during the surgical procedure. (**A**) The wound is dried with sterile gauze. (**B**) A small amount of UV-PMA (approximately 1 mL) is placed on a sterile silicone spatula. (**C**) UV-PMA is directly applied to the surgical incision in all four directions. (**D**) UV-PMA glue is polymerized with a UV lamp placed at a maximal distance of 10 cm from the wound. The total polymerization time is 60 s. (**E**) Final appearance of the surgical wound after two layers of UV-PMA glue. The total polymerization time is 120 s.

**Figure 2:**
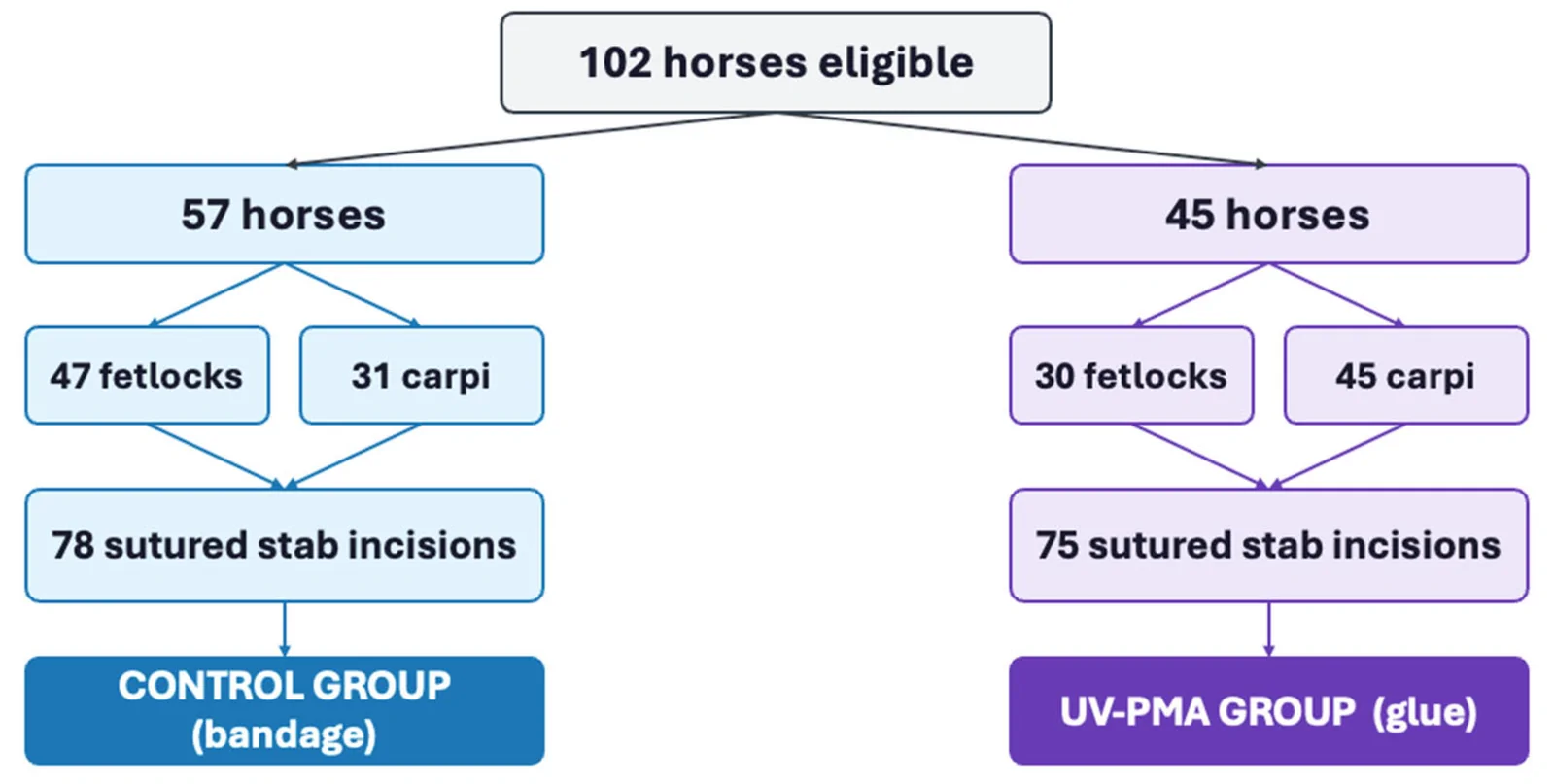
Chart showing patient enrollment and the final number of surgical incisions in each group. The surgical incisions in the control group received bandages while the horses in the clinical group only received UV-PMA glue on their surgical incisions.

**Figure 3:**
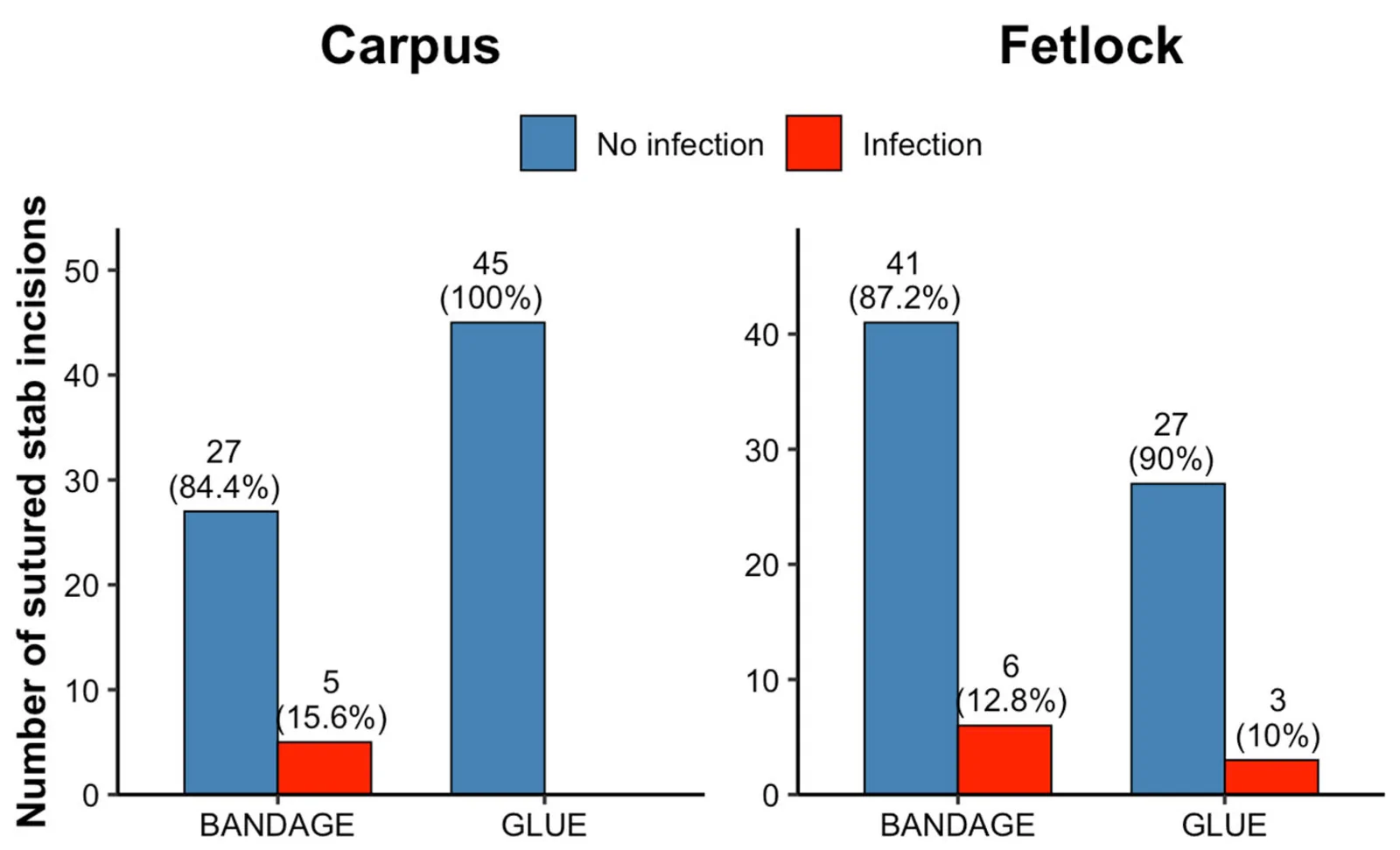
Postoperative surgical wound infections according to anatomical location and treatment group. Number of infected (in red) and non-infected (in blue) surgical wounds at the fetlock and carpus level in the bandage and UV-PMA glue groups. Labels indicate the number and percentage of incisions within each treatment group.

**Table 1:** Distribution of variables between the bandage and glue groups. The continuous variables are expressed as median (Q1, Q3) and compared with a Wilcoxon rank-sum test; the categorical variables are expressed as counts (%) and compared using a Chi-squared test or Fisher’s exact test as appropriate. * Represents *p*-value < 0.05 and is considered statistically significant.

VARIABLE		BANDAGE (n = 78)	GLUE (n = 75)	*p*-Value
Age (days)		58 (44,223)	127 (59,343)	0.007 *
Sex	Female	47 (60%)	40 (53%)	0.404
	Male	31 (40%)	35 (47%)	
Breed	Thoroughbred	54 (69%)	49 (65%)	0.909
	Standardbred	19 (24%)	19 (25%)	
	Arabians	3 (3.8%)	4 (5.3%)	
	Warmblood	2 (2.6%)	3 (4.0%)	
Joint	Carpus	31 (40%)	45 (60%)	0.015 *
	Fetlock	47 (60%)	30 (40%)	
Deviation	Varus	32 (41%)	36 (48%)	0.400
	Valgus	46 (59%)	39 (52%)	

**Table 2:** Distribution of infected surgical wounds among angular limb deformity and joints in both groups.

		BANDAGE (N = 78)		GLUE (N = 75)	
		Infected	Non-Infected	Infected	Non-Infected
**FETLOCK**	Varus	3 (3.8%)	15 (19.2%)	2 (2.7%)	14 (18.7%)
	Valgus	3 (3.8%)	26 (33.3%)	1 (1.3%)	13 (17.3%)
**CARPUS**	Varus	2 (2.6%)	12 (15.4%)	0 (0%)	20 (26.7%)
	Valgus	2 (2.6%)	15 (19.2%)	0 (0%)	25 (33%)

## Data Availability

The raw data supporting the conclusions of this article will be made available by the authors on request.

## Abbreviations

The following abbreviations are used in this manuscript:

ALD	Angular limb deformity
SSI	Surgical site infection
UV-PMA	Ultraviolet-polymerizable methacrylate adhesive
